# Evaluation of bacterial adherence and biofilm development on an anodized stainless-steel surface for the prevention of osteosynthesis-associated infections

**DOI:** 10.5194/jbji-10-581-2025

**Published:** 2025-12-09

**Authors:** Marina Medel-Plaza, María Angeles Arenas, John J. Aguilera-Correa, Amber De Bleeckere, Aranzazu Mediero, Ignacio García, Juan J. De Damborenea, Jaime Esteban, Tom Coenye, Ana Conde

**Affiliations:** 1 Department of Clinical Microbiology. IIS-Fundación Jiménez Díaz, UAM, Madrid, Spain; 2 CIBERINFEC-CIBER de Enfermedades Infecciosas, Instituto de Salud Carlos III, Madrid, Spain; 3 National Centre of Metallurgical Research, CENIM-CSIC, Madrid, Spain; 4 Laboratory of Pharmaceutical Microbiology, Ghent University, Ghent, Belgium; 5 Joint and Bone Research Unit, IIS-Fundación Jiménez Díaz, UAM, Madrid, Spain

## Abstract

**Background**: Implant-associated infections remain a major challenge in orthopaedic surgery. This study aimed to evaluate the anti-adherent and anti-biofilm properties of a novel anodized 316L stainless-steel (A 316L SS) surface against common pathogens in osteosynthesis-associated infections (OAIs). **Methods**: Bacterial adherence and biofilm formation of *Staphylococcus aureus*, *Staphylococcus epidermidis*, *Enterococcus faecalis*, *Cutibacterium acnes*, *Escherichia coli*, and *Pseudomonas aeruginosa* were assessed on A 316L SS and non-anodized 316L stainless steel (Ref 316L SS). Adherence was evaluated after 90 min using fluorescence microscopy. Biofilm development was examined after 24–48 h in synthetic synovial fluid (SSF) using colony counts and scanning electron microscopy (SEM). **Results**: A 316L SS significantly reduced bacterial adherence and surface coverage for all species tested compared to Ref 316L SS. In biofilm assays, A 316L SS exhibited notable anti-biofilm properties, with significantly reduced biofilm formation for all species. *E. faecalis* and *C. acnes* also showed lower planktonic bacterial counts. Imaging confirmed decreased bacterial presence and extracellular matrix on A 316L SS. **Conclusions**: A 316L SS shows strong anti-adherent and anti-biofilm properties against common orthopaedic pathogens, even under in vivo-like conditions. This surface modification strategy holds significant potential for reducing implant-associated infections and warrants further investigation for clinical applications.

## 1 Introduction

Prosthetic joint infections (PJIs) are major complications of orthopaedic surgery due to treatment difficulties with current antibiotics (Davidson et al., 2019). They affect 1 %–2 % of primary arthroplasty procedures, causing high morbidity and costs (Izakovicova et al., 2019). Osteosynthesis-associated infections (OAIs) constitute a significant concern in trauma surgery, potentially impeding bone healing and increasing amputation risk (Garrigós et al., 2023; Wong et al., 2020). The main challenge is microorganisms forming antibiotic-resistant biofilms on implant surfaces (Giannitsioti et al., 2022). *S. aureus* and coagulase-negative staphylococci are the primary pathogens, with increasing involvement of Gram-negative bacteria (Deva et al., 2013). Bacterial biofilms form through adherence and maturation into communities within an extracellular matrix, influenced by implant properties and host factors (Benito et al., 2019). Therefore, surface modification strategies may prevent implant-associated infections.

Metallic biomaterials, primarily stainless steel (SS), cobalt-based alloys, and titanium, are used in surgical implants for their mechanical strength and biocompatibility (Amirtharaj Mosas et al., 2022). In particular, 316L SS is common in orthopaedic plates and prostheses (Amirtharaj Mosas et al., 2022). However, all implants can be colonized by bacteria, creating challenges in preventing and treating infections. Various strategies have been investigated to address this challenge, including antibacterial coatings and surface modifications (Arteaga-Hernandez et al., 2021; Martín-García et al., 2023). Recent advances have led to innovative approaches in implant surface engineering, including nanostructured surfaces that inhibit bacterial adherence, antimicrobial agents in implant coatings, and smart anti-bacterial surfaces (Aguilera-Correa et al., 2019; Conde et al., 2024; Lozano et al., 2015). While titanium anodization has been extensively studied, SS is not commonly anodized due to technical challenges and the need for organic electrolytes (Aguilera-Correa et al., 2019; Martín-García et al., 2023; Perez-Jorge et al., 2017; Pérez-Jorge et al., 2012). Therefore, using anodized SS for antibacterial surfaces represents a promising approach (Conde et al., 2024; González et al., 2021).

The in vivo environment around prosthetic implants affects biofilm formation, and using in vivo-like media enables more clinically relevant biofilm models (Guzmán-Soto et al., 2021). Synovial fluid (SF) promotes bacterial aggregation in PJIs, facilitating bacterial–host protein interactions that enhance antibiotic tolerance (Knott et al., 2021). While these mechanisms are well studied for *S. aureus*, other pathogens like Enterobacterales also form biofilm aggregates (Macías-Valcayo et al., 2021; Staats et al., 2022). Examining these mechanisms using in vitro synovial fluid models provides insights into antimicrobial tolerance and highlights the need for targeted therapies (De Bleeckere et al., 2024).

Therefore, this study evaluated the anti-adherent and anti-biofilm properties of a novel anodized surface modification of 316L SS against common PJI and OAI pathogens. To bridge laboratory research and clinical applications, we assessed biofilm development using synthetic synovial fluid (SSF) as an in vivo-like medium (De Bleeckere et al., 2024). This approach provides insights into strategies to mitigate bacterial colonization and biofilm formation on orthopaedic implants, thereby addressing a critical challenge in infection management.

## 2 Material and methods

### 2.1 Material and surface modification

Disc samples with a diameter of 15 mm and a thickness of 3 mm were prepared from a commercial 316L SS cold-drawn bar (16.52 wt % Cr, 10.31 wt % Ni, 1.55 wt % Mn, 2.02 wt % Mo, 0.46 wt % Si, 0.020 wt % C, bal. Fe) following a previously described methodology (Conde et al., 2024).

The surface of the samples was ground using SiC abrasive paper, ranging from 200 to 3000 grit, and subsequently polished with 3-micron diamond paste. The samples were rinsed and cleaned with distilled water and ethanol before being dried in a stream of warm air.

The anodizing process was conducted in a two-electrode electrochemical cell, with a platinum foil of 2.25 cm^2^ used as the counter electrode and the 316L SS disc used as the working electrode. Only one side of the disc, with an area of 1.77 cm^2^, was anodized in an electrolyte composed of ethylene glycol (EG) containing 0.1 M NH_4_F and 0.1 M H_2_O, under no agitation conditions. The anodizing process was performed at a constant voltage and a temperature of 5 ± 1 °C. The voltage was applied in ramp mode at a rate of 1 V s^−1^ up to 50 V, which was kept constant for 15 min. The samples were then immersed in a saturated CaCO_3_ solution to remove fluorides, followed by cleaning with distilled water, rinsing with ethanol, and drying in a warm air stream.

### 2.2 Surface characterization

Surface characterization was performed as previously described (Conde et al., 2024). The nanostructure of the anodic oxide layer was examined using a field emission gun scanning electron microscope (FEG-SEM), Hitachi S 4800 J, equipped with energy-dispersive X-ray spectroscopy (EDX). The stoichiometric composition of the oxide films was further determined by Rutherford backscattering spectrometry (RBS), using He^+^ ions with 3.035 MeV energy (the resonant energy for ^16^O ( α , $\alpha_{0}$ )^16^O reaction), produced by the van de Graff accelerator at the Centre for Micro Analysis of Materials (CMAM), UAM, Madrid. The incident ion beam, with a diameter of 1 mm, was normal to the specimen surface with a dose of 10 µC , and scattered ions were detected by a fix detector at an angle of 170°. Data analysis was carried out using the SIMNRA software.

X-ray diffraction analysis was performed using a Bruker D8 Advance X-ray diffractometer with a Co anode, operating in grazing incidence at a fixed angle of 2°.

The surface roughness of the samples before and after anodizing was characterized by a Sensofar Pl µ 2300 optical imaging profiler with a 20x EPI magnification objective. Surface roughness measurements were conducted over an area of 557 × 398 µm ^2^.

### 2.3 Bacterial strains

All strains were obtained from the American Type Culture Collection (ATCC). Six strains of bacterial species frequently found as pathogens in OAIs and PJIs were selected. Among them, four were Gram-positive bacteria, including *S. aureus* ATCC 29213, *Staphylococcus epidermidis* ATCC 12228, *Enterococcus faecalis* ATCC 29212, and *Cutibacterium acnes* ATCC 6919, while two were Gram-negative bacteria, *Escherichia coli* ATCC 25922 and *Pseudomonas aeruginosa* ATCC 27853. All the strains were stored at - 80 °C.

### 2.4 Bacterial adherence

The bacterial adherence experiments on the non-anodized and anodized 316L SS (Ref 316L SS and A 316L SS, respectively) samples were performed following the methodology previously described (Conde et al., 2024), with the difference that the temperature during the 90 min incubation period was conducted at 37 °C (Sect. S1.1 in the Supplement).

### 2.5 Biofilm growth in SSF

Each bacterial strain was cultivated overnight in brain heart infusion (BHI) liquid broth (Oxoid, Hampshire, UK) at 37 °C in 5 % CO_2_ atmosphere. *C. acnes* was anaerobically incubated for 48 h (Anaerocult A mini, Merck, Darmstadt, Germany). Before starting, the 316L SS samples were disinfected and then vortexed at 3500 rpm for 15 s in distilled water (Merck KGaA, Darmstadt, Germany) and placed into a sterile 12-well plate (SPL Life Sciences, Pocheon-si, Gyeonggi-do, Korea). The strains were cultured in brain heart infusion broth (Merck KgaA, Darmstadt, Germany) at 37 °C for 24 h (40 h for *C. acnes*), then centrifuged at 3500 rpm for 10 min. The supernatant was removed, and the pellet was washed three times with sterile saline. Subsequently, for each species, ∼ 10^6^ CFU mL^−1^ in SSF were inoculated into each well. SSF was prepared and validated as an in vivo-like medium (Bleeckere et al., 2023) (see formula in Sect. S1.2). Subsequently, the plate was incubated for 24 h at 37 °C in a 5 % CO_2_ atmosphere. *C. acnes* was incubated for 48 h under anaerobic conditions (Anaerocult A Mini). After incubation, the samples were washed thrice with saline to eliminate planktonic bacteria. Subsequently, mature biofilm was scrapped from the disc surface with sterile wooden sticks, which were then sonicated in a 50 mL Falcon^™^ conical tube (Thermo Fisher Scientific, United States) with 10 mL of 0.9 % NaCl for 5 min (Branson Ultrasonic, Emerson Electric Co., Missouri, USA) (Esteban et al., 2008). This sonicated saline was 1:10 serially diluted with saline, and the biofilms were quantified using the drop plate method (Herigstad et al., 2001). After incubation of the plates, colony-forming units (CFUs) were counted and CFU cm^−2^ was calculated. The remaining supernatant volume in each well after removing the SS samples for washing was used to estimate the concentration of planktonic bacteria (CFU mL^−1^), following the same plating method as described in Sect. S1.1. The variable total CFU was estimated by combining biofilm-associated and planktonic bacteria (see formula in Sect. S1.2). Each experiment was performed using three biological replicates and two technical replicates per biological replicate ( n= 6 per strain and condition).

Representative micrographs of each strain on different surface finishes were taken by field emission gun scanning electron microscope (FEG-SEM) (Fig. 6). To do so, after the biofilm growth on the surface of the metallic samples in SSF and the corresponding washing steps, each sample was placed in a well from a 12-well plate and immersed in 2 mL of hexamethyldisilazane (HMDS) for 15 min at room temperature under a chemical hood. After that incubation, HMDS was removed and discarded, and each sample was dried completely under the chemical hood. All the samples were observed by using an APREO 2S-THERMOFISHER-CYRO-FESEM (Thermo Scientific) equipped with a high-resolution QUORUM PP3010T station (QUORUM Technologies). Operating conditions were low-vacuum, 40 Pa, 5 kV, secondary and backscattered camera detectors, with a working distance of 5–8 mm.

### 2.6 Ion release assessment

Both Ref 316L SS and A 316L SS samples were vortexed for 15 s at 3000 rpm in distilled water for injection (B. Braun, Melsungen, Germany). These samples were incubated in a 6-well plate with 5 mL of 0.9 % NaCl saline solution at 37 °C and 5 % CO_2_ for 90 min. At least 1 mL of saline was taken at 90 min to estimate the iron (Fe), chromium (Cr), nickel (Ni), and fluoride (F^−^) by inductively coupled plasma mass spectrometry in Reference Laboratory (Barcelona, Spain). Concentration was determined using an ion-selective electrode in Reference Laboratory S.A. (Hospitalet de Llobregat, Spain). This experiment was performed with 200 µg m^−1^ deferoxamine (DFX) in the medium to stabilize the cations and in triplicate.

Similarly, cation release (Fe, Cr, and Ni) assays were performed after 24 and 72 h using a 50 % SSF/PBS dilution following the same procedure previously described (Sise et al., 2025). Each sample was immersed in 2 mL of the corresponding dilution, following the same protocol detailed in Sect. 2.5. A 50 % dilution of SSF in PBS was used. This concentration has been demonstrated to provide protective effects comparable to those of 100 % SF under in vitro conditions, due to the sufficient presence of lubricin and hyaluronic acid, with no significant differences in surface roughness observed (Sise et al., 2025). Water contact angles were measured using distilled water and a Theta Attension optical tensiometer (KSV Instruments), equipped with an automatic multi-liquid dispenser and a monochromatic cold light source. The contact angles were calculated using the Young–Laplace drop profile fitting method. Each reported value represents the average of three measurements taken at distinct locations on the sample surface. The volume of each water droplet was 3 µL . For each droplet, 30 frames were analysed to estimate the mean contact angle. All measurements were performed in ambient air at 298 K. These experiments were performed in triplicate.

### 2.7 Cytotoxicity

Cytocompatibility of Ref 316L SS and A 316L SS surfaces was evaluated though an indirect cell viability. The MC3T3-E1 pre-osteoblastic cell line (ATCC, USA) was cultured in α -minimum essential medium ( α -MEM; Thermo Fisher Scientific) supplemented with 10 % fetal bovine serum (FBS) and 1 % penicillin–streptomycin. Cells were kept at 37 °C in a humidified atmosphere with 5 % CO_2_. For the cytotoxicity assessment, cells were seeded in 96-well plates at a concentration of 1 × 10^4^ cells per well and allowed to adhere overnight. Each well was incubated with 200 µL of each type of medium for 48 h: control medium (C) (medium with no metal sample), Ref 316L SS medium (medium where a Ref 316L SS was incubated in a 50 mL tube containing 5 mL of complete culture medium for 72 h at 37 °C and 5 % CO_2_), and A 316L SS (medium where A 316L SS was incubated in a 50 mL tube containing 5 mL of complete culture medium for 72 h at 37 °C and 5 % CO_2_). Cell viability was evaluated using MTT assay following manufacture protocol. A growth control (C) was included, consisting of cells cultured in standard medium without any metallic surface. Ref 316L SS and A 316L SS conditions were compared to C and among them to assess any potential cytotoxic effects linked to each finishing surface. The experiment was performed by using three replicates of each material/condition and using 8 wells per replicate ( n= 24 wells per material/condition). Cytotoxicity results were evaluated according to the ISO 10993-5 standard. According to this standard, cytotoxicity can be classified into four grades: Grade 0 (None), no reduction in cell growth; Grade 1 (slight), No more than 20 % growth inhibition; Grade 2 (mild), no more than 50 % inhibition of cell growth is observed; Grade 3 (moderate), no more than 50 % growth inhibition is observable; Grade 4 (severe), almost complete or total destruction of the cell layers.

### 2.8 Statistical analysis

For surface characterization, data were processed following ISO 25178 using a Gaussian L filter ( λc = 80 × 80 µm ). Three regions per condition were analysed. Normality was assessed using a Shapiro–Wilk test (Origin). ANOVA with Levene's test was used to evaluate differences. Sa values are expressed as mean ± SD.

Statistical analyses were performed using R (version 4.5.1; R Foundation for Statistical Computing, Vienna, Austria) and GraphPad Prism 10 (GraphPad Software, San Diego, CA, USA). For adherence assays (planktonic bacterial load) and ion release experiments, normality was assessed using the Shapiro–Wilk test. Depending on data distribution, results were expressed as mean ± SD and compared using a Student's t test or as median (IQR) and compared using the Wilcoxon rank–sum test. Mixed-effects analysis was applied (*lme4, ImerTest*) to the adherence (excluding the planktonic bacteria variable), biofilm, and cytotoxicity assays, with surface type as a fixed effect and experiments as a random effect. Surface type was coded with the variable A 316L SS as the reference level. Therefore, positive coefficients indicate higher values on the untreated 316L SS compared to the anodized surface. Significance was set at p<0.05 .

## 3 Results

### 3.1 Surface characterization

**Figure 1:**
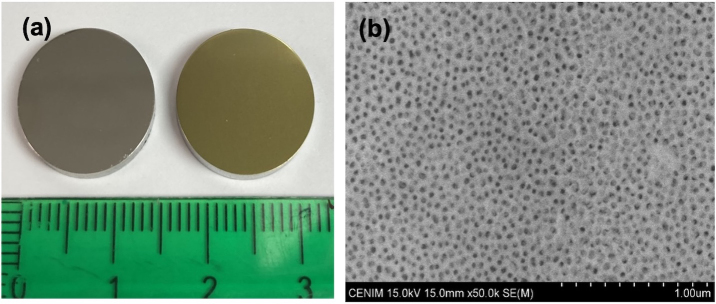
Surface finish of 316L SS samples before and after the anodizing process **(a)** and SEM image of nanoporous anodic layer growth on A 316L SS **(b)**.

316L is an austenitic SS with a lower chromium and higher nickel and molybdenum content compared to 304L SS. During the anodizing process, 316L samples underwent a colour change from a shiny grey metallic appearance towards a gold-like finish and increased surface roughness (Fig. 1a). The mean surface roughness (Sa) of the 316L before anodizing was 13.8 ± 0.6 and 126 ± 13 nm after anodizing. The SEM analysis of the anodized 316L SS samples reveals the nanoporous morphology of the anodic film with average pore diameter ∼ 25 ± 3 nm (Fig. 1B). The EDX analysis revealed an F content of 32.2 at. % and Fe, O, and Cr contents of 31.3 at. %, 14.1 at. %, and 9.2 at. %, respectively, along with lower contents of Ni (4.5 at. %) and Mo (0.6 at. %).

**Figure 2:**
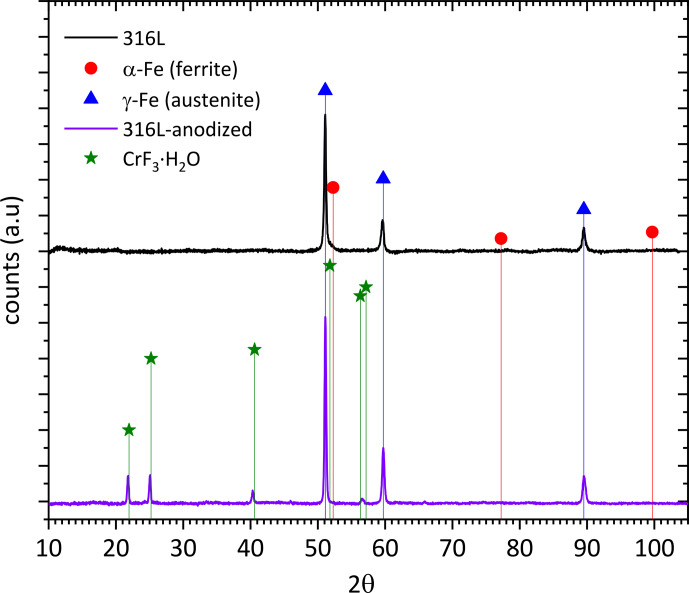
X-ray diffractograms corresponding to A 316L SS and Ref 316L SS.

The comparison of the XRD diffractograms for both A 316L SS and Ref 316L SS is shown in Fig. 2. The stronger peaks corresponding to γ -Fe (austenite) are clearly observed in both samples, whereas smaller additional peaks corresponding to hydrated chromium F^−^ are only observed in the anodized samples.

**Figure 3:**
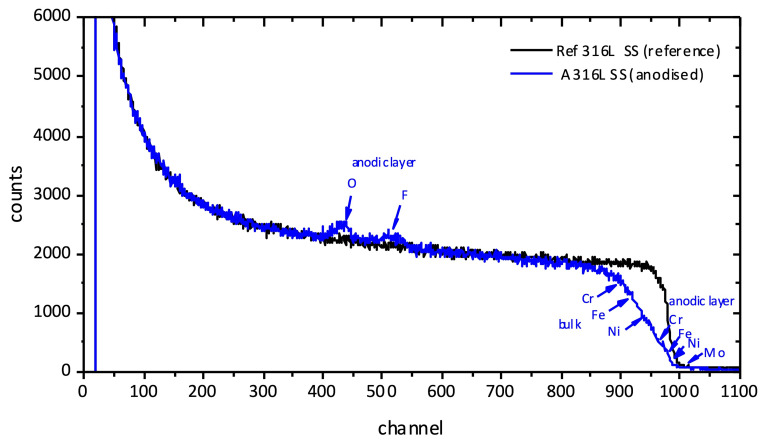
Comparison of the RBS spectra of A 316L SS and Ref 316L SS.

The composition and thickness of the anodic films were also examined by RBS. Figure 3 compares the RBS spectra corresponding to the A 316L SS and Ref 316L SS samples. The yields of fluorine and oxygen in the anodic film appear separately from the Cr, Fe, and Ni yields both in the bulk and in the anodic film.

The average molecular composition of the anodic layer estimated from the RBS analysis is gathered in Table 1. The anodic film consists of an inner F-enriched layer of ∼ 61 nm thickness and an outer layer of ∼ 240 nm thickness. The outer layer is primarily composed of Fe, Cr, and NiF_2_ and lower contents of Cr oxide and mixed Fe molybdenum oxide.

**Table 1:** Average molecular composition of the anodic film formed on A 316L SS.

	Layer	Average molecular	Thickness
		composition	(nm)
			
Nanoporous	Outer	FeF ${}_{1.7\;}\cdot 0.26$ CrF_3_ ⋅	
anodic		0.15NiF_2_ ⋅ 0.26 Cr_2_O_3−*x*_	
film		⋅ 0.04FeMoO_4_	240.95
	F-enriched	FeF_1.16_	61.04
	Total thickness		301.99

### 3.2 Bacterial adherence

**Figure 4:**
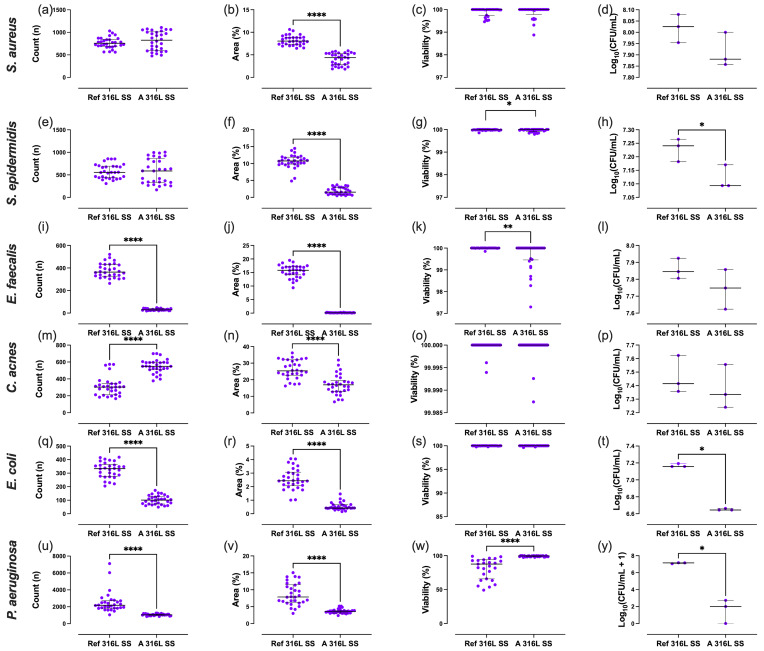
Quantification of bacterial parameters for *S. aureus* **(a–d)**, *S. epidermidis* **(e–h)**, *E. faecalis* **(i–l)**, *C. acnes* **(m–p)**, *E. coli* **(q–t)**, and *P. aeruginosa* **(u–y)**. Aggregate count **(a, e, i, m, q, u)**, percentage of surface coverage **(b, f, j, n, r, v)**, adhered bacterial viability **(c, g, k, o, s, w)**, and planktonic bacterial load **(d, h, l, p, t, y)** were analysed after exposure to anodized A 316L SS or Ref 316L SS. ^*^ p<0.05 ; ^***^ p<0.001 ; ^****^ p<0.0001 .

Differences in outcomes between the two SS sample types were evaluated (Fig. 4). For *S. aureus* and *S. epidermidis*, the surface area covered on A 316L SS was significantly reduced by 45.6 % and 85.7 %, respectively (Fig. 4b and 4f). *E. faecalis* showed a notable 92.2 % decrease in aggregate count on A 316L SS (Fig. 4i), along with a 99.2 % reduction in surface coverage (Fig. 4j). For *C. acnes*, the aggregate count increased by 80.6 % on A 316L SS (Fig. 4m), while surface coverage decreased by 33.3 % (Fig. 4n). In the case of *E. coli*, there was a significant 69.7 % reduction in aggregate count (Fig. 4q), an 82.6 % decrease in surface coverage (Fig. 4r), and a 69.5 % reduction in planktonic CFU mL^−1^ on A 316L SS (Fig. 4t). For *P. aeruginosa*, the aggregate count decreased by 52.0 % (Fig. 4u), surface coverage decreased by 55.0 % (Fig. 4v), and there was a pronounced 99.9 % reduction in planktonic CFU mL^−1^ (Fig. 4y).

In *S. aureus*, anodization significantly reduced the surface area covered ( Δ= + 4.13; 95 % CI 3.70–4.57; p<0.0001 ) (Fig. 4b). *S. epidermidis* also exhibited a significantly larger area covered on the Ref 316L SS surface ( Δ= + 8.78; 95 % CI 7.98–9.58; p<0.0001 ), along with a modest increase in viability ( Δ= + 0.025; 95 % CI 0.001–0.050; p= 0.043) (Fig. 4f and g). For *E. faecalis*, the Ref 316L SS supported higher CFU counts ( Δ= + 346.4; 95 % CI 324.6–368.1; p<0.0001 ) (Fig. 4i) and a larger area covered ( Δ= + 15.3; 95 % CI 14.5–16.0; p<0.0001 ) (Fig. 4j), with a modest rise in viability ( Δ= + 0.34; 95 % CI 0.11–0.58; p= 0.005) (Fig. 4k). In *C. acnes*, CFU counts were significantly lower on Ref 316L SS ( Δ= - 237.7; 95 % CI - 281.1 to - 194.2; p<0.0001 ) (Fig. 4m), whereas the surface area covered was greater ( Δ= + 9.19; 95 % CI 6.56–11.83; p<0.0001 ) (Fig. 4n). *E. coli *showed markedly higher CFU counts ( Δ= + 222.8; 95 % CI 199.0–246.7; p<0.0001 ) (Fig. 4q) and area covered ( Δ= + 2.03; 95 % CI 1.73–2.33; p<0.0001 ) (Fig. 4r) on Ref 316L SS. *P. aeruginosa* formed substantially more CFU ( Δ= + 1458; 95 % CI 1007–1910; p<0.0001 ) (Fig. 4u) and area covered ( Δ= + 5.04; 95 % CI 3.93–6.16; p<0.0001 ) (Fig. 4v) on Ref 316L SS, but with significantly lower viability ( Δ= - 15.7; 95 % CI - 21.1 to - 10.3; p<0.0001 ) (Fig. 4w). Regarding planktonic bacteria concentration, *S. epidermidis* (Fig. 4h), *E. coli* (Fig. 4t), and *P. aeruginosa* (Fig. 4y) exhibited reductions of 22.4 %, 69.5 %, and 55.0 % on A 316L SS compared to Ref 316L SS, respectively.

Representative fluorescence microscopy images of each bacterial species and surface finishing are shown in Fig. S1 in the Supplement.

### 3.3 Biofilm development in SSF

**Figure 5:**
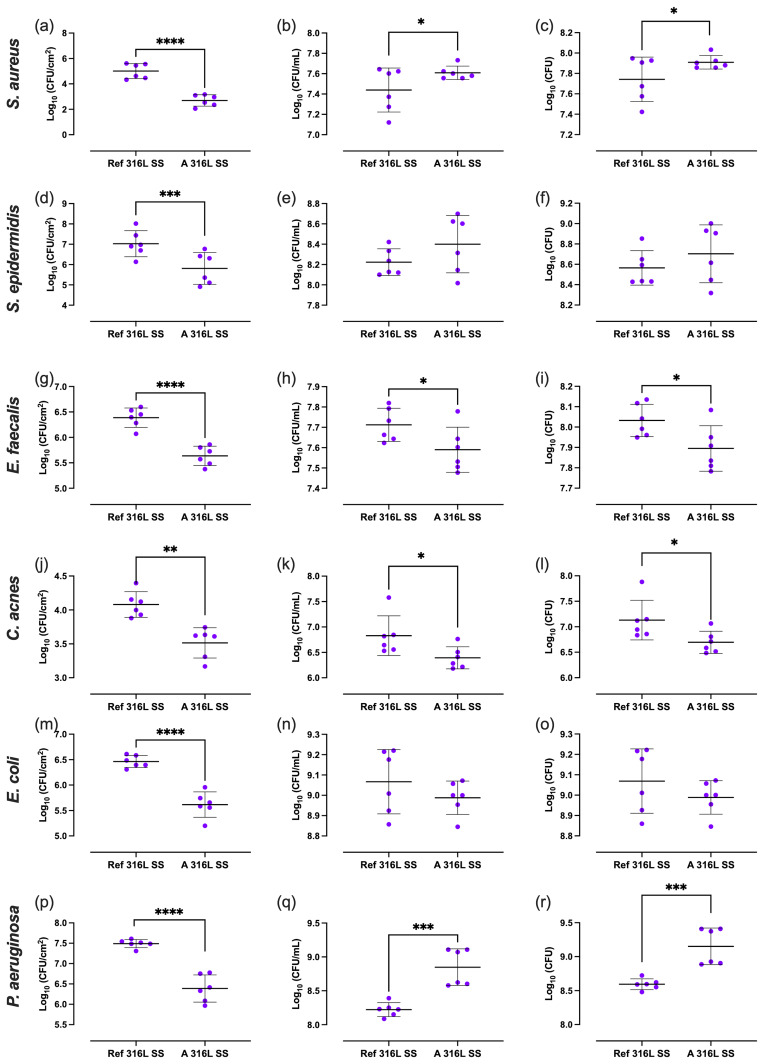
Biofilm-covered surface **(a, d, g, j, m, p)**, planktonic bacteria **(b, e, h, k, n, q)**, and total bacterial load **(c, f, i, l, o, r)** of *S. aureus* **(a–c)**, *S. epidermidis* **(d–f)**, *E. faecalis* **(g–i)**, *C. acnes* **(j–l)**, *E. coli* **(m–o)**, and *P. aeruginosa* **(p–r)** on Ref 316L SS and 316L S). ^*^ p<0.05 ; ^***^ p<0.001 ; ^****^ p<0.0001 .

Differences in outcomes between the two SS sample types were evaluated (Fig. 5). Regarding biofilm-covered surface, all bacterial species exhibited lower CFU cm^−2^ values on the A 316L SS compared to on the Ref 316L SS, with significant reductions of 99.5 %, 97.7 %, 82.3 %, 72.8 %, 85.7 %, and 92.1 % for *S. aureus* (Fig. 5a), *S. epidermidis* (Fig. 5d), *E. faecalis* (Fig. 5g), *C. acnes* (Fig. 5j), *E. coli* (Fig. 5m), and *P. aeruginosa* (Fig. 5p), respectively. In terms of the concentrations of planktonic bacteria in the supernatant in contact with each surface, a significant reduction of 24.6 % and 58.6 % was observed on the A 316L SS for *E. faecalis* (Fig. 5h) and *C. acnes* (Fig. 5k), respectively. Regarding the total bacterial load, significant differences were found between both samples for *E. faecalis* and *C. acnes*, with reductions of 27.2 % (Fig. 5i) and 58.8 % (Fig. 5l), respectively. Conversely, *P. aeruginosa* showed higher CFU mL^−1^ and total CFU on the A 316L SS compared to the Ref 316L SS, with increases of 322.6 % (Fig. 5q) and 270.3 % (Fig. 5r), respectively.

Mixed-effects analysis revealed clear differences between A 316L SS and Ref 316L SS (Fig. 5). For *S. aureus*, biofilm-associated CFUs (CFU cm^−2^) were significantly reduced on the anodized surface ( Δ= + 2.31 log; 95 % CI 1.72 – 2.91; p<0.0001 ) (Fig. 5a). *S. epidermidis* also showed increased biofilm formation on Ref 316L SS ( Δ= + 1.22 log; 95 % CI 0.56–1.87; p= 0.0065) (Fig. 5d). In *E. faecalis*, both CFU cm^−2^ ( Δ= + 0.75 log; 95 % CI 0.54–0.97; p<0.0001 ) and total CFUs ( Δ= + 0.14 log; 95 % CI 0.03–0.24; p= 0.032) were higher on Ref 316L SS (Fig. 5g, i). *C. acnes* exhibited consistent differences, with higher biofilm ( Δ= + 0.56 log; 95 % CI 0.33–0.80; p= 0.0015), planktonic ( Δ= + 0.44 log; 95 % CI 0.10–0.77; p= 0.033), and total CFUs ( Δ= + 0.44 log; 95 % CI 0.11–0.77; p= 0.033) on Ref 316L SS (Fig. 5j–l). *E. coli* also presented significantly higher CFU cm^−2^ on Ref 316L SS ( Δ= + 0.85 log; 95 % CI 0.63–1.07; p<0.0001 ) (Fig. 5m). Finally, *P. aeruginosa* displayed a distinct profile: biofilm CFU cm^−2^ were significantly greater on Ref 316L SS ( Δ= + 1.10 log; 95 % CI 0.82–1.38; p<0.0001 ), while planktonic ( Δ= - 0.63 log; 95 % CI - 0.82 to 0.44; p= 0.0002) and total CFUs ( Δ= - 0.56 log; 95 % CI - 0.74 to - 0.37; p= 0.0004) were lower compared to Ref 316L SS.

**Figure 6:**
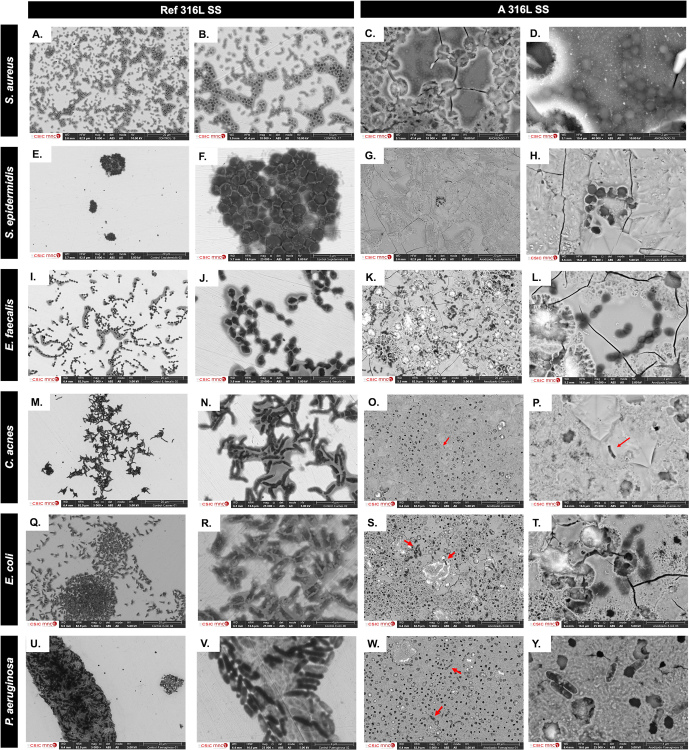
SEM images comparing bacterial colonization on the non-anodized 316L SS (Ref 316L SS) **(a, b, e, f, i, j, m, n, q, r, u, v)** and anodized 316L SS (A 316L SS) **(c, d, g, h, k, l, o, p, s, t, w, y)** of *S. aureus* **(a–d)**, *S. epidermidis* **(e–h)**, *E. faecalis* **(i–l)**, *C. acnes* **(m–p)**, *E. coli* **(q–t)**, and *P. aeruginosa* **(u–y)**.

SEM images comparing microbial colonization on reference and anodized surfaces are shown in Fig. 6. On Ref 316L SS, *S. aureus* and *S. epidermidis* form multilayered grape-like clusters within a matrix (Fig. 6a, b, e, f), while, on A 316L SS, they appear as isolated cocci with a minimal matrix (Fig. 6c, d, g, h). *E. faecalis* forms chain groups within a matrix on Ref 316L SS (Fig. 6i, j) but shows dispersed chains without a matrix on A 316L SS (Fig. 6k, l). *C. acnes* exhibits stratified bacterial layers in the matrix on Ref 316L SS (Fig. 6m, n), with sparse presence on A 316L SS (Fig. 6o, p). *E. coli* forms compact biofilm on Ref 316L SS (Fig. 6q, r), with detached cells on A 316L SS (Fig. 6s, t). *P. aeruginosa* aggregates in a matrix on Ref 316L SS (Fig. 6u, v) but disperses with a minimal matrix on A 316L SS (Fig. 6w, y). These images demonstrate different bacterial attachment patterns between A 316L SS and Ref 316L SS.

### 3.4 Ion release

#### 3.4.1 Fe, Cr, Ni, and F in 0.9% NaCl saline solution

**Figure 7:**
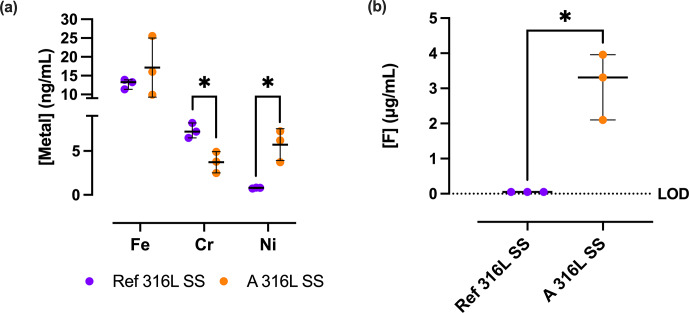
Fe, Cr, Ni **(a)**, and F^−^ **(b)** release from the non-anodized 316L SS (Ref 316L SS) (purple) and anodized 316L SS (A 316L SS) (orange) after 90 min of incubation in saline supplemented with 200 µg mL^−1^ deferoxamine. ^*^ p<0.05 . Limit of detection (LOD) = 0.1 µg mL^−1^.

The release of Fe, Cr, and Ni cations (Fig. 7) from the Ref 316L SS and A 316L SS surfaces exhibited notable differences. Cr release was significantly lower in A 316L SS (372.0 ± 121.1 ng mL^−1^) compared to Ref 316L SS (730.0 ± 85.4 ng mL^−1^) ( p= 0.0173). Ni release was significantly higher in A 316L SS (572.3 ± 181.0 ng mL^−1^) than in Ref 316L SS (78.3 ± 5,7 ng mL^−1^) ( p= 0.0418). However, no statistically significant differences were observed in Fe release. Similarly, the F^−^ release analysis revealed a statistically significant increase in F^−^ ion concentration on the A 316L SS compared to the Ref 316L SS ( p= 0.0318). For Ref 316L SS, the median F^−^ content is negligible, since this condition does not contain F^−^, whereas the A 316L SS exhibited a median release of 3.31 (2.10–3.96) µg mL^−1^.

#### 3.4.2 Fe, Ni, and Cr in 50 % SSF/PBS

**Figure 8:**
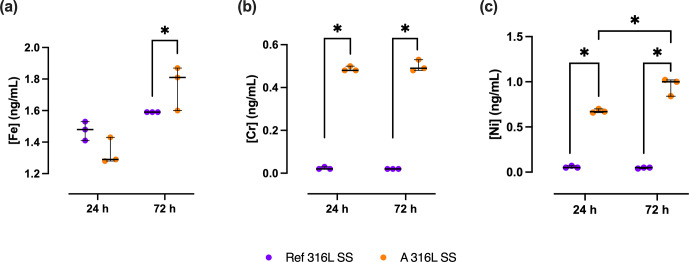
Metal ion release of Fe **(a)**, Cr **(b)**, and Ni **(c)** from A 316L SS (orange) and Ref 316L SS (purple) surfaces after 24 and 72 h in 50 % SSF/PBS. ^*^ p<0.05 .

The release of Fe, Cr, and Ni cations from A 316L SS and Ref 316L SS surfaces in 50 % SSF/PBS showed clear differences (Fig. 8).

After 72 h, Fe release from A 316L SS was 17.6 % (0.63 %–22.3 %) higher than that from Ref 316L SS ( p= 0.05). At 24 h, Cr release from A 316L SS was 24.0 (23.0–23.0)-fold higher than from Ref 316L SS ( p<0.05 ). After 72 h, Cr release from the anodized surface remained significantly elevated, corresponding to a 25.5-fold increase compared to the reference ( p<0.05 ). Ni release followed a similar pattern, being 12.4 (12.2–13.0)-fold higher in A 316L SS than in Ref 316L SS after 24 h ( p<0.05 ) and rising to 19.0 (15.8–19.4)-fold higher after 72 h ( p<0.05 ). Comparing Ni release between the two time points, A 316L SS surface released 49.3 % (25.4 %–52.3 %) more Ni at 72 h relative to 24 h ( p<0.05 ).

The wettability showed no change either among the different surface finishings or between the different times (24 and 72 h) (Fig. S2).

#### 3.4.3 Cytotoxicity

**Figure 9:**
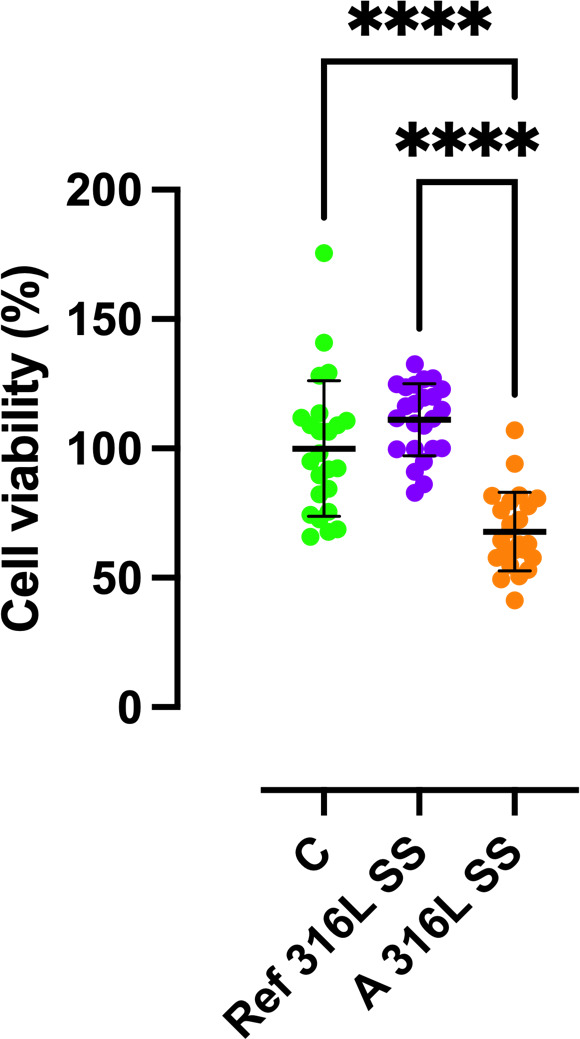
Percentage of viable MC3T3-E1 cells obtained for the positive control growth (C) (green), Ref 316L SS (purple), and A 316L SS (orange) surfaces after 24 h of incubation. ^****^ p<0.0001 .

The mixed-effects analysis revealed a significant influence of the material on cell viability (Fig. 9). The estimated mean viability for A 316L SS surface was 31.12 % (95 % CI: 21.7 %–42.5 %) lower than that observed for the growth control ( p<0.0001 ). This means that the cytotoxicity of A 316L SS surface can be classified as Grade 2 (mild), since no more than 50 % inhibition of cell growth was observed. Similarly, Ref 316L SS conditions exhibited a 43.2 % (95 % CI: 31.8 %–53.7 %) higher viability compared to the anodized surface ( p<0.0001 ).

## 4 Discussion

Anodizing valve metals forms barrier or duplex oxide layers with nanoporous morphology, depending on electrolyte composition and electrical parameters. Anodizing iron-based alloys requires organic electrolytes with controlled water additions, as growth mechanism depends on Fe-base alloy composition (Fadillah et al., 2020). Studies on nanoporous anodic F^−^ films on 304L SS revealed high F^−^ incorporation with antimicrobial properties (Conde et al., 2024), and anodizing 316L SS reduced bacterial attachment across common pathogens from PJIs and OAIs (Benito et al., 2019).

The literature shows differential bacterial adherence reductions across species. Erdogan and Ercan (2023) reported 71 % decreased *S. aureus* adherence using EG monobutyl solution with perchloric. Jang et al. (2018) attributed anti-fouling to 200 nm nanoarchitectures, whereas our work shows effective properties with 10–40 nm nanopores. The anti-adherent effect of modified surfaces stems from changes in roughness, hydrophobicity, and chemistry through anodization (Conde et al., 2024). These modifications hinder bacterial attachment during the “window of opportunity” phase (Deva et al., 2013).

Biofilm formation studies on medical implants are essential for preventing implant-associated infections. While traditional in vitro models use conventional growth media, physiologically relevant models are being explored, such as synthetic cystic fibrosis medium for *P. aeruginosa* (De Bleeckere et al., 2023). For PJI, models using human synovial fluid (Staats et al., 2022) or SSF (Stamm et al., 2022) have been developed. De Bleeckere et al. (2024) created an SSF model for studying biofilm development and antibiotic tolerance in PJI under joint-like conditions, which was adopted in this study. Our study first investigates A 316L SS against PJI and OAI pathogens using SSF, mimicking physiological conditions. One of the bacterial behaviours observed was that all bacteria tested formed biofilm-like aggregates with variations in shape and size, except for *P. aeruginosa*, while *E. faecalis* and *C. acnes* formed the fewest aggregates due to slower growth (De Bleeckere et al., 2024). Some bacteria actually thrive in SSF; for instance, *S. aureus* aggregates through fibrinogen and fibronectin receptors, which are key components of our SSF (Staats et al., 2022), and even biofilm-deficient *S. epidermidis* forms aggregates with downregulated agr genes (Stamm et al., 2022).

Contrary to the pattern observed in other species, the total and planktonic counts of *S. epidermidis* and *P. aeruginosa* increased in SSF when comparing A 316L SS to Ref 316L SS surfaces (Fig. 5). This behaviour may stem from genotypic adaptation due to exposure to SF, which can drive the selection of phenotypes better suited to survival in joint-like environments (Steixner et al., 2021).

Among the *Staphylococci*, *S. epidermidis* exhibits a remarkable adaptive capacity that allows it to persist under hostile conditions, such as those simulated by the SSF (Perez and Patel, 2015). In these environments, the bacterium can modulate the expression of virulence-related genes and biofilm-associated pathways, including the *icaADBC* operon global regulators such as *sarA* and *ArIRS* (Bottagisio et al., 2020; Burke et al., 2024). This behaviour could enhance survival and persistence in nutrient-limited, protein-rich environments, explaining the slight, non-significant increase observed in both planktonic and total viable counts for *S. epidermidis*. This suggests that the trend is more likely due to the adaptive interaction between the bacteria and the SSF medium rather than the intrinsic effect of A 316L SS surface itself.

We found that A 316L SS released significantly higher levels of Cr, Ni, and F ions than Ref 316L SS, which likely exerted cytotoxic pressure. However, *P. aeruginosa* possesses robust metal homeostasis systems, including high-affinity Fe acquisition through siderophores such as pyoverdine and pyochelin, detoxification of non-Fe metals via siderophore-mediated binding, and active metal efflux pumps such as CzcCBA (Hassen and Abbassi, 2025). Moreover, *P. aeruginosa* can utilize Fe^2+^ through the FeoB system, bypassing siderophore-mediated Fe^3+^ acquisition, and efficiently utilize SSF components such as glutamine, alanine, and arginine as nutrient sources (Cornelis and Dingemans, 2013; McGill et al., 2021). All these factors, together with the fact that, under high metal concentrations, *P. aeruginosa* increases adaptive responses (Braud et al., 2009), may explain its rise in both planktonic and total CFU in A 316L SS. However, further studies would be required.

The anti-biofilm effect of A 316L SS might be multifaceted, and it is crucial to differentiate between the contributions of surface nanostructure and compositional alterations induced by anodization. In fact, in our investigation, we created a surface with 32 at. % fluorine concentration, potentially influencing antibacterial properties. This aligns with Perez-Jorge et al. (2017), who demonstrated antibacterial properties in anodic layers with an F^−^ content ranging from 4.2 at. % to 12 at. %. Anodization incorporates the anion and enhances its release into the environment, thereby contributing to the bactericidal effect (Perez-Jorge et al., 2017). F^−^ toxicity primarily arises from interference with enzymatic processes and intracellular accumulation in bacterial cells (Banerjee et al., 2024). However, some bacterial species have evolved resistance mechanisms, like F-specific efflux pumps, to counteract its toxic effects, such as *E. coli* and *P. aeruginosa*, with *crcB* encoding an F-specific transporter (Chellaiah et al., 2021). In addition, *P. aeruginosa* shows broader F^−^ tolerance by increasing antioxidant enzyme activity and biofilm formation (Singh et al., 2023).

Beyond F^−^, the cytotoxic effects of metallic cations have been widely studied due to their influence on bacterial viability and host cells (Begg, 2019). Our findings revealed that Fe was released from A 316L SS in the highest concentration, followed by Ni (Fig. 6). Bacteria have developed mechanisms to acquire Fe from their surroundings, such as siderophores like enterobactin in *E. coli* and pyoverdine or pyochelin in *P. aeruginosa* (Cunrath et al., 2015). The increased release of Ni could suggest a competitive interaction with Fe for siderophore binding, affecting bacterial metabolism. Ni toxicity involves metal displacement in metalloproteins, enzyme inhibition, and the induction of oxidative stress (Begg, 2019). Furthermore, the presence of chelators in the SSF medium, such as citrate, histidine, cysteine, and glutamate, enhances Ni solubility and availability, increasing bacterial uptake through nonspecific transporters (Sreeshma and Sudandiradoss, 2021). *E. faecalis* and *C. acnes* utilize other metal transporters (e.g. MntABC, EfeUOB) and alternative chelators, respectively, for Ni uptake (Brüggemann et al., 2021; Brunson et al., 2023).

Cr and Ni, while potentially toxic to bacteria, may also exert cytotoxic effects on host cells (Zhong et al., 2024). However, the degree of toxicity depends on multiple factors, including ion concentration, exposure time, and the alloy composition (Liu et al., 2023). Under typical clinical or simulated physiological conditions, the cytotoxic response is considered mild or negligible (Osman et al., 2022), which is consistent with the findings of the present study. The cell viability results obtained indicate that both the control and the reference alloy supported higher cellular viability than A 316L SS, although they maintained viability cell levels consistent with a mild cytotoxic effect according to ISO 10993-5 standards (UNE-EN ISO 10993-5:2009 Evaluación biológica de productos sani., 2025), confirming the overall biocompatibility of A 316L SS.

This study has some limitations. Although the use of an SSF model provided a physiologically relevant in vitro environment, the results cannot be directly extrapolated to clinical outcomes. Moreover, only a limited number of reference bacterial strains were tested; including a broader range of clinical and multi-drug-resistant isolates in future studies would provide a more comprehensive assessment of the antimicrobial performance of A 316L SS.

## 5 Conclusions

This study demonstrates A 316L SS's potential in reducing bacterial adherence and biofilm formation in osteosynthesis implant infections. The anodization process created a nanoporous surface with F^−^, showing significant reductions in bacterial colonization across pathogens. The use of SSF provided physiologically relevant insights into biofilm development. While this is promising, further research is needed to evaluate long-term efficacy and in vivo performance. This work contributes valuable knowledge towards developing improved biomaterials for orthopaedic surgery.

## Supplement

10.5194/jbji-10-581-2025-supplement The supplement related to this article is available online at https://doi.org/10.5194/jbji-10-581-2025-supplement.

## Data Availability

The data supporting the findings of this study are available from the corresponding author upon reasonable request.
