# Supplementary material for: Evaluation of bacterial adherence and biofilm development on an anodized stainless-steel surface for the prevention of osteosynthesis-associated infections
Source: J Bone Jt Infect. 2025 Dec 9;10(6):581–95. doi: 10.5194/jbji-10-581-2025 (PMC12753106; doi:10.5194/jbji-10-581-2025)
Supplement: The supplement related to this article is available online at https://doi.org/10.5194/jbji-10-581-2025-supplement. [file jbji-10-581-2025-supplement.pdf]

Supplement of J. Bone Joint Infect., 10, 581–595, 2025
https://doi.org/10.5194/jbji-10-581-2025-supplement
© Author(s) 2025. CC BY 4.0 License.

Supplement of

Evaluation of bacterial adherence and biofilm development on an anodized stainless-steel surface for the prevention of osteosynthesis-associated infections

Marina Medel-Plaza et al.

Correspondence to: Ana Conde (a.conde@cenim.csic.es)

The copyright of individual parts of the supplement might differ from the article licence.

S1. Supplementary methods

S1.1. Bacterial adherence

Prior to each experiment, all 316L SS samples were rinsed and vortexed for 15 s at 300 rpm in pure distilled water (B. Braun, Germany). Each bacterial strain was cultured in tryptic soy broth (bioMérieux, Marcy-l'Étoile, France) at 37ºC for 24 h. After incubation, bacterial cultures were centrifuged at 3500 rpm for 10 minutes, and the supernatant was discarded. The pellet was washed three times with sterile 0.9% NaCl saline solution. Subsequently, bacteria were resuspended and diluted in 0.9% NaCl to achieve a bacterial concentration of $\sim 10^8$ CFU/mL (0.5 McFarland). A volume of 5 mL of this bacterial suspension was statically incubated with the 316L SS samples in sterile untreated 6-wells plates (Thermo Fisher Scientific, MA, USA) at 37ºC for 90 min.(Arenas et al., 2013) After incubation, the samples were washed three times with saline to remove non-adhered bacteria, as previously described.(Pérez-Jorge et al., 2012) The metallic samples were then stained using the LIVE/DEAD™ BacLight™ bacterial viability kit (Thermo Fisher Scientific, MA, USA) and rinsed with sterile water .(Boulos et al., 1999) Approximately 10 images of different fields at 40× magnification were taken for each sample using a DM 2000 fluorescence microscope (Leica Microsytems, Wetzlar, Germany). Each image was randomly taken from each sample and covered an area of 167.5 µm × 223.15 µm (total area per image of 37,386.6 µm^2). All images were taken under the same microscopy settings (315.5–463.1-ms exposure time, 5.5× optical gain, 1.50 saturation level, and gamma of 10.00). The aggregates count, percentage of the surface covered by adhered bacteria, along the proportions of live and dead bacteria were calculated using ImageJ software (National Institutes of Health, Bethesda, MD, USA).(Aguilera-Correa et al., 2019) Additionally, after the 316L SS samples were carefully removed from each well and transferred for the subsequent washing steps, the remaining supernatant, which had been in contact with the samples for 90 min, was collected to determine the concentration of planktonic bacteria (CFU/mL). Serial dilutions were plated(Herigstad et al., 2001) on tryptic soy agar supplemented with 5% sheep blood (bioMérieux, Marcy-l'Étoile, France) for *S. aureus, S. epidermidis* and *E. faecalis.* Schaedler agar (bioMérieux, Marcy-l'Étoile, France) in anaerobiosis was used for *C. acnes* and MacConkey agar (bioMérieux, Marcy-l'Étoile, France) for *E. coli* and *P. aeruginosa*. This experiment was performed using three biological replicates for each strain and type of material.

S1.2. Biofilm development in SSF

SSF composition: (75.2 g/L $Na_2HPO_4 \cdot 2H_2O$, 30 g/L KH_2PO_4, 5 g/L NaCl, 5 g/L NH_4Cl, 1 g/L Glucose, 0.2465 $MgSO_4$, 0.0441 g/L $CaCl_2$, 0.498 g/L $FeCl_3$, 0.084 g/L $ZnCl_2$, 17 g/L $CuCl_2 \cdot 2H_2O$, 47.6 g/L $CoCl_2 \cdot 6H_2O$, 6.2 g/L H_3BO_3, 198 g/mL $MnCl_2 \cdot 4H_2O$, 0.014 g/L tyrosine, 0.038 g/L glutamic acid, 5 g/L alanine, 1.2 g/L L-arginine HCl, 1.2 g/L L-asparagine monohydrate, 0.88 g/L aspartic acid, 2 g/L L- cysteine HCl monohydrate, 7.6 g/L glutamine, 2.9 g/L glycine, 1.8 g/L histidine, 1.3 g/L isoleucine, 3.5 g/L lysine, 0.43 g/L methionine, 2.7 g/L leucine, 1.6 g/L phenylalaline, 2.8 g/L proline, 2 g/L Serine, 1.9 g/L threonine, 1.4 g/L tryptophan, 2.7 g/L valine, 0.002 g/L nicotinic acid, 2 g/L thiamine, 0.002 g/L calcium pantothenate, 0.0001 g/L biotin, 0.024 g/L citric acid, 0.1 g/L, 25 g/L bovine serum albumin, 3 g/L hyaluronic acid, and 0.100 g/L fibrinogen)

Calculation of total CFU: the variable total CFU was estimated by summing the bacteria forming the biofilm (CFU/cm^2) and the planktonic bacteria (CFU/mL) using the following formula: total CFU = (1,767cm^2 × CFU/cm^2) + (2 mL × *y* CFU/mL).

S2. Supplementary results

S2.1. Bacterial adherence

LIVE/DFEAD fluorescence microscopy images comparing bacterial adherence are shown in Fig. S1.

Staphylococcus aureus forms dense clusters on Ref 316L SS (Fig. S1a), while A 316L SS (Fig. S1b) shows significantly fewer viable bacteria. *Staphylococcus epidermidis* shows substantial adherence with uniform green fluorescence on Ref 316L SS (Fig. S1c), but exhibits reduced bacterial presence on A 316L SS (Fig. S1d). *Enterococcus faecalis* extensively colonizes Ref 316L SS with clustered bacteria (Fig. 1Se), while A 316L SS (Fig. S1f) shows minimal adherence. *Cutibacterium acnes* form large, dense aggregates on Ref 316L SS (Fig. S1g), whereas A 316L SS (Fig. 1Sh) shows fewer bacteria in small clusters. *Echerichia coli* forms a uniform layer on Ref 316L SS (Fig. S1i), with reduced attachment on A 316L SS (Fig. S1j). *Pseudomonas aeruginosa* creates a compact layer with dead cells on Ref 316L SS (Fig. S1k), while on A 316L SS (Fig. S1l), bacteria are dispersed with reduced adherence.

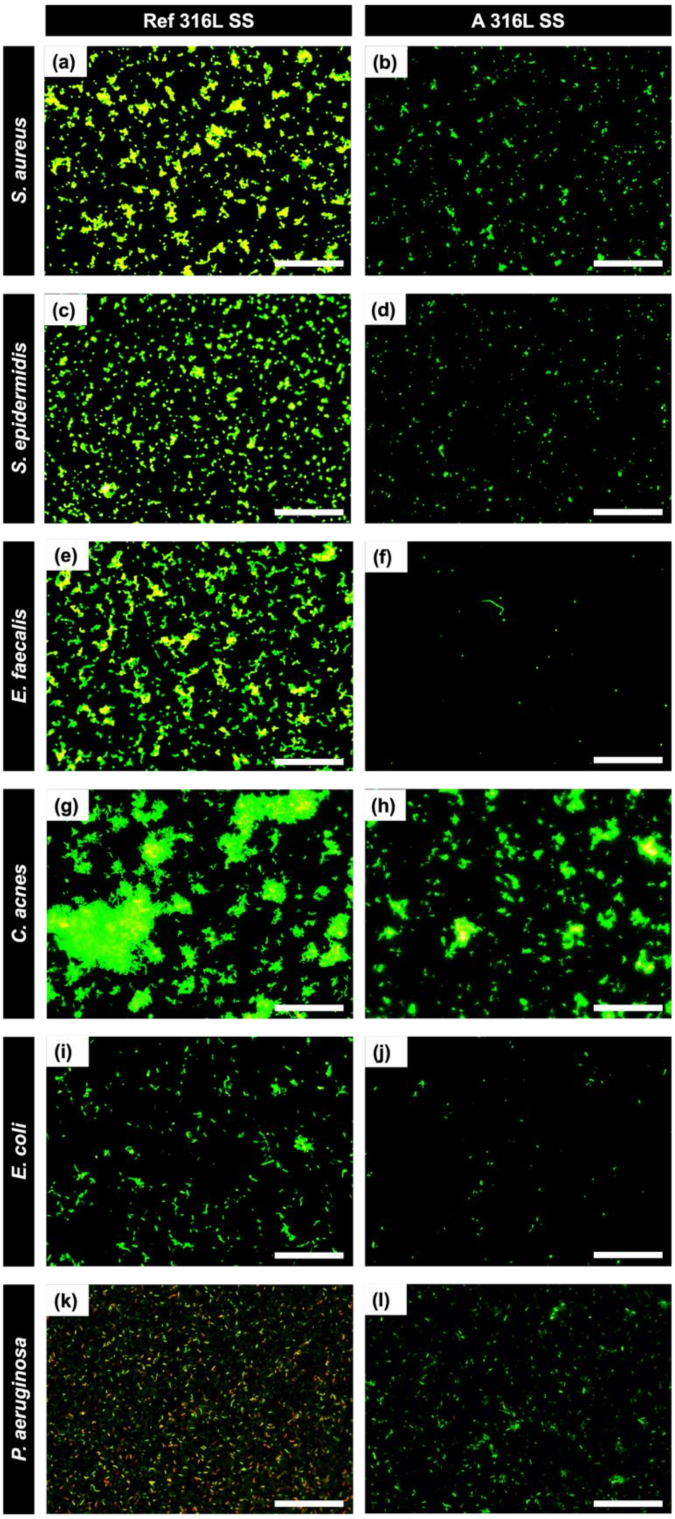

Figure S1. Images of *S. aureus* (a,b), *S. epidermidis* (c, d), *E. faecalis* (e, f), *C. acnes* (g, h), *E. coli* (i, j), and *P. aeruginosa* (k, l) adhered to non-anodised 316L SS, (Ref 316L SS) (a, b, e, g, i, k) and anodised 316L surface (A 316L SS) (b, d, f, h, j, l). Images were obtained using a DM 2000 Fluorescence microscope. Scale bar: 50 µm.

S2.2. Hydrophobicity assay

The static contact angle analysis revealed no significant differences in surface wettability between Ref 316L SS and A 316L SS at either time point (24 h and 72 h).

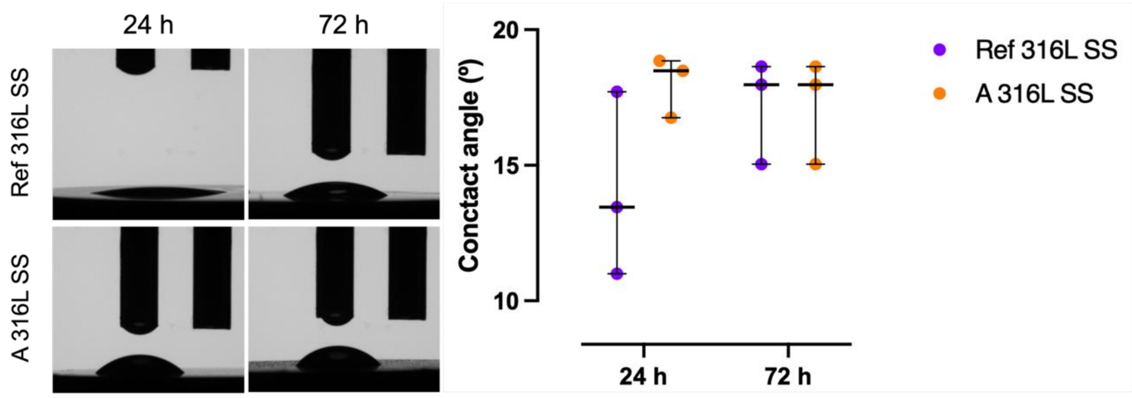

Figure S2. Static contact angle measurements of Ref 316L SS (purple) and A 316L SS (orange) surfaces after 24 h and 72 h.
